# A Systematic Review of Clinical Prediction Rules to Predict Hospitalisation in Children with Lower Respiratory Infection in Primary Care and their Validation in a New Cohort

**DOI:** 10.1016/j.eclinm.2021.101164

**Published:** 2021-10-18

**Authors:** Dermot M Wildes, Master Chisale, Richard J Drew, Peter Harrington, Chris J Watson, Mark T Ledwidge, Joe Gallagher

**Affiliations:** a gHealth Research Group, UCD Conway Institute, School of Medicine, University College Dublin, Ireland; b Biological Science Department, Faculty of Science, Technology & Innovations, Mzuzu University, Malawi; c Irish Meningitis and Sepsis Reference Laboratory, Temple Street, Dublin 2; d Wellcome-Wolfson Institute for Experimental Medicine, Queen's University Belfast, Northern Ireland

**Keywords:** Paediatrics, Respiratory Medicine, Infectious disease, Global health, Pneumonia

## Abstract

**Background:** Our goal was to identify existing clinical prediction rules for predicting hospitalisation due to lower respiratory tract infection (LRTI) in children in primary care, guiding antibiotic therapy. A validation of these rules was then performed in a novel cohort of children presenting to primary care in Malawi with World Health Organisation clinically defined pneumonia.

**Methods:** MEDLINE & EMBASE databases were searched for studies on the development, validation and clinical impact of clinical prediction models for hospitalisation in children with lower respiratory tract infection between January 1^st^1946-June 30^th^ 2021. Two reviewers screened all abstracts and titles independently. The study was conducted in accordance with the Preferred Reporting Items for Systematic Reviews & Meta-Analyses guidelines.

The BIOTOPE cohort (BIOmarkers TO diagnose PnEumonia) recruited children aged 2-59 months with WHO-defined pneumonia from two primary care facilities in Mzuzu, Malawi. Validation of identified rules was undertaken in this cohort.

**Findings:** 1023 abstracts were identified. Following the removal of duplicates, a review of 989 abstracts was conducted leading to the identification of one eligible model. The CHARMS checklist for prediction modelling studies was utilized for evaluation. The area under the curve (AUC) of the STARWAVe rule for hospitalisation in BIOTOPE was found to be 0.80 (95% C.I of 0.75-0.85). The AUC of STARWAVe for a confirmed diagnosis of bacterial pneumonia was 0.39 (95% C.I 0.25-0.54).

**Interpretation:** This review highlights the lack of clinical prediction rules in this area. The STARWAVe rule identified was useful in predicting hospitalisation from bacterial infection as defined. However, in the absence of a gold standard indicator for bacterial LRTI, this is a reasonable surrogate and could lead to reductions in antibiotic prescription rates, should clinical impact studies prove its utility. Further work to determine the clinical impact of STARWAVe and to identify diagnostic tests for bacterial LRTI in primary care is required.

**Panel: Research in context**

**Evidence before this study**

Prior to this review, there had been no published systematic review of clinical prediction models for predicting hospitalisation due to bacterial lower respiratory tract infection in children in primary care. Clinical prediction models combine variables derived from the history, examination and basic investigations to guide clinicians by providing them with a probability of a target diagnosis, facilitating improved clinical decision making and decreased intervention. OVID MEDLINE & EMBASE were searched for all studies pertaining to the development, validation and clinical impact of clinical prediction models for bacterial causes of lower respiratory tract infection in children published between 1946 and quarter-2, 2021. There was no language restriction. Models pertaining to hospitalised patients, adult or neonatal populations only, or those that used single predictors or investigations beyond the remit of primary care were excluded.

**Added value of this study**

This systematic review identifies the only eligible model available and performs a validation study in a novel cohort. This shows a paucity of clinical prediction rules in this area. The STARWAVe rule was shown to be a useful tool for predicting hospitalisation in both Europe and African cohorts. However, it was poor at identifying bacterial infection as defined in the BIOTOPE study. In the absence of a gold standard indicator for bacterial LRTI, we believe that this is a reasonable surrogate and could lead to significant reductions in antibiotic prescription rates, should clinical impact studies prove its utility.

**Implications of all the available evidence**

This article demonstrates the global applicability of the STARWAVe rule to predict hospitalisation in children with lower respiratory tract infection. There is a need for further work to develop and determine the impact of clinical prediction rules in primary care for hospitalisation from bacterial lower respiratory tract infection and their role in improving appropriate antimicrobial prescribing.

Alt-text: Unlabelled box

## 1 Introduction

Pneumonia is the greatest single cause of paediatric mortality of all diseases [1]. Children are perceived as a vulnerable population, and it has been acknowledged that primary care clinicians have a tendency to provide early therapeutic intervention, despite a very low level of clinical suspicion for the presence of a bacterial aetiology with respiratory tract infections, in an endeavour to minimise the risk of potential hospitalisation [2,3]. This practice of defensive medicine to avoid hospitalisation often manifests in the over-prescription of antibiotics. Poor antibiotic stewardship is a driving force in the development of antimicrobial resistance, manifesting as a decrease in available treatment options, in conjunction with the failure of previously reliable treatments, in the context of both ordinary and more severe infections.

The vast majority of antibiotic prescribing takes place in the setting of primary care, accounting for 74% of all antibiotic prescriptions in the United Kingdom in 2016 [4]. In an international context, this figure is likely to be even higher; countries that lack the secondary and tertiary care infrastructure of their more developed counterparts are more reliant on primary care. The insufficient number of appropriately trained and well-supported physicians in less developed settings, the lack of tools to differentiate bacterial from viral infection and higher morbidity and mortality in low-income countries may lead primary care clinicians to be excessively conservative in their therapeutic decision making [4,5].

Clinical prediction rules are tools which combine variables derived from the history, examination and basic investigations to guide clinicians by providing them with a probability of a target diagnosis [6]. Used correctly, clinical prediction rules can serve to reassure clinicians in their decision to avoid therapeutic intervention, adopting a 'watch and wait' approach [7].

This aim of this study was to identify existing clinical prediction rules for predicting hospitalisation secondary to lower respiratory tract infection in children in primary care, with the aim to guide clinicians in their decision to provide antibiotic therapy and to undertake validation of these rules in a novel cohort of children presenting in primary care in Malawi with World Health Organisation (WHO) clinically defined pneumonia.

## 2 Methods

The systematic review was conducted in accordance with the PRISMA (Preferred Reporting Items for Systematic Reviews and Meta-Analyses) guidelines [8]. The Checklist for Critical Appraisal and Data Extraction for Systematic reviews of prediction modelling studies (CHARMS) checklist for the appraisal of prediction models was also utilised in this systematic review [9].

### 2.1 Search Strategy

The review question and design was framed using the CHARMS checklist for systematic reviews of prediction models (see supplemental component) [9] A systematic search strategy was then constructed for use in MEDLINE OVID and EMBASE. (Details on the search syntax can be found in Table 1). We searched for studies on the development, validation and clinical impact of clinical prediction models for causes of lower respiratory tract infection in children published between 1946 and quarter-2, 2021. There was no language restriction. We also consulted the reference lists of included articles, the supplemental file of an international register of clinical prediction rules (CPRs) [10] and experts in the area for further articles**.**

**Table 1:** Search strategy

OVIDMEDLINE	Search Terms
1	(respiratory tract infection or respiratory infection* or rti or lrti or lri or chest infection* or cough or dyspnoea or congestion or lung consolidation or pneumonia or difficult breath* or respiration disorder*).mp. [mp=title, abstract, original title, name of substance word, subject heading word, floating sub-heading word, keyword heading word, protocol supplementary concept word, rare disease supplementary concept word, unique identifier, synonyms]
2	(child* or schoolchild* or preschool* or paediatric* or paediatric* or infant or infancy*).mp. [mp=title, abstract, original title, name of substance word, subject heading word, floating sub-heading word, keyword heading word, protocol supplementary concept word, rare disease supplementary concept word, unique identifier, synonyms]
3	(Model* or Predict* or Decision*OR score* or rul*).mp. [mp=title, abstract, original title, name of substance word, subject heading word, floating sub-heading word, keyword heading word, protocol supplementary concept word, rare disease supplementary concept word, unique identifier, synonyms]
4	(primary care or family practice or general practice or family medicine or community healthcare or primary healthcare or ambulatory care).mp. [mp=title, abstract, original title, name of substance word, subject heading word, floating sub-heading word, keyword heading word, protocol supplementary concept word, rare disease supplementary concept word, unique identifier, synonyms]
5	1 and 2
6	4 and 5
7	3 and 6
*	The asterisk (*) represents any group of characters, including no character
""	Only finds articles with this phrase

### 2.2 Eligibility Criteria

Inclusion Criteria:

- Population: Paediatric human patients (aged 2-59 months).
- Multivariable models for the likelihood of hospitalisation due to lower respiratory tract infection or to guide initiation of antimicrobial therapy in primary care.
- Outcome measure: Hospitalisation secondary to lower respiratory tract infection or prescription of antibiotics for lower respiratory tract infection.
- Setting of care: hospital outpatient, emergency department or primary care (studies of clinical prediction rules conducted in non–primary care settings were eligible for inclusion, only if they involved investigative tests available routinely in primary care.

Exclusion Criteria:

- Studies which outline models designed solely for the diagnosis of patients admitted to hospital.
- Studies which outline models designed for use in patients outside the 2-59 month demographic or where this age group could not be identified as a sub-group.
- Studies which outline models that utilise variables beyond the scope of primary care (e.g. radiology, biochemical investigations requiring a laboratory).
- Studies which used single predictors only, as they are prone to reporting overly optimistic findings.

The scope of this review focussed our approach on screening for existing clinical prediction rules addressing the likelihood of hospital admission due to lower respiratory tract infection in children, with the aim to guide antimicrobial therapy and to undertake validation of these rules in a novel cohort. This was done by ensuring the variables utilised were easily assessable in the primary care setting. For example, variables such as symptoms, signs and pulse oximetry are easily obtained whereas items such as laboratory testing (other than point of care testing) or chest x-ray are not.

In order to enhance the international applicability of this study, broad inclusion criteria were used – thereby acknowledging the discrepancies in the availability of certain diagnostic tests to clinicians in different geographical settings.

#### 2.2.1 Critical Appraisal

Two reviewers (DW & JG) screened all titles and abstracts independently for potential inclusion to the sub-set for full-text review. Any discrepancies in opinion were settled by consensus.

Data extraction was performed using the CHARMS checklist, under the following headings: source of data; participant characteristics; predicted outcomes; candidate predictors; sample size; missing data; model development; model performance; model evaluation and results.

#### 2.2.2 External Validation

Rules were validated in the BIOTOPE cohort (BIOmarkers TO diagnose PnEumonia) which recruited children aged 2 months to 59 months with World Health Organisation defined pneumonia from two primary care facilities in Mzuzu, Northern Malawi [11,12]. The WHO algorithm indicates that all these children should have antibiotics. Clinical symptoms, signs, and examinations were obtained using systematic procedures [12].

Blood, oropharyngeal and nasopharyngeal specimens were collected to perform rapid diagnostic tests, culture and molecular tests for pathogens causing pneumonia, HIV, and malaria. A final diagnosis of bacterial, viral or undetermined aetiology pneumonia was determined using pre-determined algorithms.

#### 2.2.3 Data Analysis

For applicable rules, we retrospectively calculated the individual score or threshold of each included patient based on the model parameters. The area under the curve (AUC) of the receiver operating characteristics curve, with 95% confidence intervals (CIs) were used to compare the overall discriminative ability of the rule. Univariate and multi-variate analyses of markers of severity, including those found in STARWAVe where available are also outlined in the Supplemental file accompanying this paper.

All calculations including statistical analysis were carried out using R Language version 3.2.3.

##### 2.2.3.1 Ethics Statement:

The protocol and related documents were approved by the National Health Science Research Committee of Malawi and the Mzuzu Central Hospital Research Committee. Patients received timely treatment and supportive care as required. The study was conducted in compliance with the ethical standards as outlined by two ethics committees and was aligned with the values outlined by the Declaration of Helsinki. (National Health Research Committee of Malawi Ethics Approval #15/11/1532).

#### 2.2.4 Role of Funding Source

This study was funded in whole or in part by the support of the Bill & Melinda Gates Foundation (Investment ID: OPP1139557). The foundation was not involved in study design, data collection, analysis, interpretation or drafting of this report.

## 3 Results

**Figure 1:**
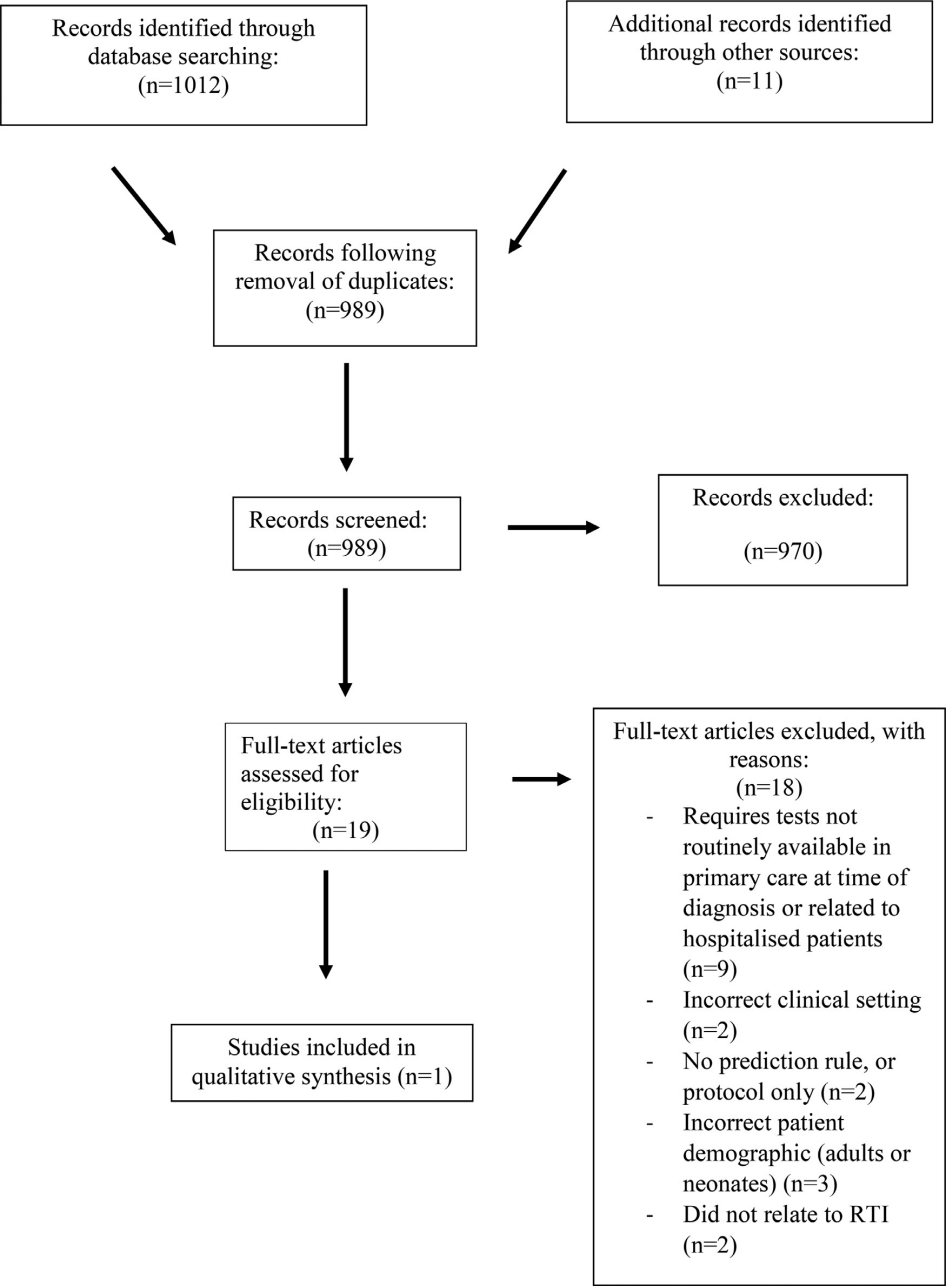
PRISMA Diagram. *See table 3 for full exclusion list.

A PRISMA flow diagram of the search strategy is shown (Figure 1). 1023 abstracts were identified and following the removal of duplicates, a review of 989 abstracts was conducted leading to the identification of one eligible clinical rule to predict hospitalisation secondary to LRTI in the community – the STARWAVE rule [13].

The STARWAVe rule was derived from a general practice cohort in the United Kingdom recruiting between 2011 to 2013. It had 33 candidate predictors involving demographics and clinical symptoms and signs. There were seven final variables. It used hospitalisation within 30 days as the outcome, a surrogate for the need for antibiotic prescription. The study involved derivation and internal validation using bootstrapping.

### 3.1 Bias

Those assessing outcomes were blinded to the predictors. Since the outcome chosen was hospitalisation within 30 days the GP who assessed the predictors would not have known the outcome unless the child was admitted on the same day, which represented 19% of total admissions. Events per variable were 78 admissions for 33 candidate variables (2.36 EPV).

### 3.2 External Validation

Validation of the STARWAVe rule was undertaken in the BIOTOPE cohort as described previously [11]. In brief, the BIOTOPE cohort consisted of 494 children with WHO clinically defined pneumonia presenting to primary care in Malawi. The median age was 18 months (IQR 10-30) and 54.8% were male. In total, 13 children had a diagnosis of bacterial pneumonia and 56 children were referred to hospital. Results of external validation revealed that the STARWAVe clinical prediction rule performs similarly in the BIOTOPE cohort when compared to its performance in the original study for hospitalisation. (Table 2). The area under the curve (AUC) of the STARWAVe rule for hospitalisation in BIOTOPE was found to be 0.8 (95% confidence interval of 0.75-0.85) comparing favourably with that of the original cohort (AUC 0.82, 95% CI 0.77-0.87). At a score of 4 or above, the STARWAVe rule had a sensitivity of 0.32 (95% CI 0.20-0.46), specificity 0.91 (95% CI 0.88-0.94) and accuracy 85% (95% CI 81-88%). However, the AUC of STARWAVe for a confirmed diagnosis of bacterial pneumonia in BIOTOPE was 0.39 (95% CI 0.25-0.54), with a sensitivity of 0.08 (95% CI 0.02-0.36), specificity 0.89 (95% CI 0.85-0.91) and accuracy 86% (95% CI 83% -89%) We also undertook further univariate and multi-variate analyses of markers of severity, including those found in STARWAVe where available (supplemental file).

**Table 2:** Risk of hospital admission and risk of bacterial pneumonia in the BIOTOPE cohort using the STARWAVe rule.

Number of predictors	Hospitalised children	Non-hospitalised children	Risk of hospital admission	Risk (95% CI)
Hospitalisation				
0 to 1	0/56 (0%)	107/438 (24.4%)	0/107 (0%)	-
2 to 3	38/56 (67.9%)	293/438 (66.9%)	38/331 (11.5%)	0.11 (0.08, 0.15)
4 or more	18/56 (32.1%)	38/438 (8.7%)	18/56 (32.1%)	0.32 (0.20, 0.46)
Total	56/56 (100%)	438/438 (100%)	56/494 (11.3%)	0.11 (0.08, 0.14)
Bacterial Pneumonia	Bacterial pneumonia present	Bacterial pneumonia absent	Risk of bacterial pneumonia	
0 to 1	5/13 (38.5%)	102/481 (22.3%)	5/107 (4.5%)	0.04 (0.01, 0.10)
2 to 3	7/13 (53.8%)	324/481 (66.7%)	7/331 (2.1%)	0.03 (0.01, 0.05)
4 or more	1/13 (7.7%)	55/481 (11.4%)	1/56 (1.8%)	0.02 (0.00, 0.10)
Total	13/13 (100%)	481/481 (100%)	13/494 (2.6%)	0.03 (0.02, 0.05)

This uses a simplified scoring system where one point is given to each predictor with no weighting to any particular predictor.

**Table 3:** Full Text Articles Excluded (with reasons):

Number	Source	Reference	Reason for exclusion
1	External	Lynch T, Platt R, Gouin S, Larson C, Patenaude Y. Can we predict which children with clinically suspected pneumonia will have the presence of focal infiltrates on chest radiographs? Pediatrics. 2004 Mar;113(3 Pt 1):e186-9. doi: 10.1542/peds.113.3.e186. PMID: 14993575.	Investigations beyond the scope of primary care - Uses chest radiograph infiltrates as a predictor.
2	External	Moreno L, Krishnan JA, Duran P, Ferrero F. Development and validation of a clinical prediction rule to distinguish bacterial from viral pneumonia in children. Pediatr Pulmonol. 2006 Apr;41(4):331-7. doi: 10.1002/ppul.20364. Erratum in: Pediatr Pulmonol. 2006 May;41(5):494. PMID: 16493666.	Investigations beyond the scope of primary care - Required the use of an FBC in its prediction method .
3	Search	Ababneh MA, Al-Azzam SI, Ababneh R, Rababa'h AM, Demour SA. Antibiotic prescribing for acute respiratory infections in children in Jordan. International Health. 2017 Mar;9(2):124-130. DOI: 10.1093/inthealth/ihx003.	No prediction rule - Lists various predictive factors, but does not collate them into a clinical prediction rule.
4	Search	Castro, A V., et al. "Additional Markers to Refine the World Health Organization Algorithm for Diagnosis of Pneumonia." Indian Pediatrics, vol. 42, no. 8, 2005, pp. 773-81.	Investigations beyond the scope of primary care - Uses chest radiograph infiltrates as a predictor.
5	Search	Feldstein DA, Hess R, McGinn T, et al. Design and implementation of electronic health record integrated clinical prediction rules (iCPR): a randomized trial in diverse primary care settings. Implement Sci. 2017;12(1):37. Published 2017 Mar 14. doi:10.1186/s13012-017-0567-y	Incorrect patient demographic - Included patients up to the age of 70 in study.
6	Search	Validation of a clinical rule to predict complications of acute cough in preschool children: a prospective study in primary care	Not confined to LRTI - Used acute cough as an inclusion criterion without other signs of lower respiratory tract infection.
		Alastair D Hay, Catharine Gorst, Alan Montgomery, Tim J Peters, Tom Fahey	
		British Journal of General Practice 2007; 57 (540): 530-537.	
7	Search	Clinical profile and predictors of severe illness in young South African infants (<60 days) Jeena, P. M.; Adhikari, M.; Carlin, J. B.; Qazi, S.; Weber, M. W.; Hamer, D. H South African Medical Journal. Suid-Afrikaanse Tydskrif Vir Geneeskunde;98(11):883-8	Incorrect patient demographic - Only included infants up to 60 days of life.
8	Search	Margolis P, Gadomski A. The rational clinical examination. Does this infant have pneumonia? JAMA. 1998 Jan 28;279(4):308-13. doi: 10.1001/jama.279.4.308. PMID: 9450716.	Incorrect patient demographic – Included patients up to the age of 19.
9	Search	Myles PR, Nguyen-Van-Tam JS, Lim WS, Nicholson KG, Brett SJ, Enstone JE, McMenamin J, Openshaw PJ, Read RC, Taylor BL, Bannister B, Semple MG. Comparison of CATs, CURB-65 and PMEWS as triage tools in pandemic influenza admissions to UK hospitals: case control analysis using retrospective data. PLoS One. 2012;7(4):e34428. doi: 10.1371/journal.pone.0034428. Epub 2012 Apr 3. PMID: 22509303; PMCID: PMC3317953.	Incorrect patient demographic – included adults along with children.
			Incorrect clinical setting – emergency medicine setting, not primary care.
10	Search	Clinical predictors of antibiotic prescribing for acutely ill children in primary care: an observational study	Not confined to lower respiratory tract infection.
		Kathryn O'Brien, Thomas Wyn Bellis, Mark Kelson, Kerenza Hood, Christopher C Butler, Adrian Edwards	
		British Journal of General Practice 2015; 65 (638): e585-e592. DOI: 10.3399/bjgp15 × 686497	
11	Search	Thompson M, Van den Bruel A, Verbakel J, Lakhanpaul M, Haj-Hassan T, Stevens R, Moll H, Buntinx F, Berger M, Aertgeerts B, Oostenbrink R, Mant D. Systematic review and validation of prediction rules for identifying children with serious infections in emergency departments and urgent-access primary care. Health Technol Assess. 2012;16(15):1-100. doi: 10.3310/hta16150. PMID: 22452986; PMCID: PMC4781278.	Incorrect clinical setting – majority of patients recruited were from hospital emergency medicine settings.
12	Search	Torres FA, Passarelli I, Cutri A, Leonardelli A, Ossorio MF, Ferrero F. Seguridad de una regla de predicción para el manejo inicial de niños con neumonía tratados en forma ambulatoria [Safety of a clinical prediction rule for initial management of children with pneumonia in an ambulatory setting]. Arch Argent Pediatr. 2010 Dec;108(6):511-5. Spanish. doi: 10.1590/S0325-00752010000600006. PMID: 21132247.	Investigations beyond the scope of primary care - Required the use of an FBC and chest radiograph in its prediction method.
13	Search	Determinants of community-acquired pneumonia in children and young adults in primary care	No prediction rule - Looked at risk factors rather than a specific clinical prediction rule.
		J. Teepe, L. Grigoryan, T. J. M. Verheij	
		European Respiratory Journal May 2010, 35 (5) 1113-1117; DOI: 10.1183/09031936.00101509	
14	Search	Redmond, N.M., Davies, R., Christensen, H. et al. The TARGET cohort study protocol: a prospective primary care cohort study to derive and validate a clinical prediction rule to improve the targeting of antibiotics in children with respiratory tract illnesses. BMC Health Serv Res 13, 322 (2013). https://doi.org/10.1186/1472-6963-13-322	Investigations beyond the scope of primary care - Employed the use of nasopharyngeal swabs and laboratory
15	External	Williams DJ, Zhu Y, Grijalva CG, et al. Predicting Severe Pneumonia Outcomes in Children. Pediatrics. 2016; 138(4):e20161019	Investigations beyond the scope of primary care - Uses laboratory/radiographic parameters
16	External	Preston Dean, Todd A Florin, Factors Associated With Pneumonia Severity in Children: A Systematic Review, *Journal of the Pediatric Infectious Diseases Society*, Volume 7, Issue 4, December 2018, Pages 323–334, https://doi.org/10.1093/jpids/piy046	Investigations beyond the scope of primary care - Uses laboratory/radiographic parameters
17	External	Edwards G, Newbould L, Nesbitt C, Rogers M, Morris RL, Hay AD, et al. (2021) Predicting poor outcomes in children aged 1–12 with respiratory tract infections: A systematic review. PLoS ONE 16(4): e0249533. https://doi. org/10.1371/journal.pone.0249533	Investigations beyond the scope of primary care - Uses laboratory/radiographic parameters
18	External	Ferrero F, Adrián Torres F, Domínguez P, Ossorio MF. Efficacy and safety of a decision rule for using antibiotics in children with pneumonia and vaccinated against pneumococcus. A randomized controlled trial. Arch Argent Pediatr. 2015 Oct;113(5):397-403. English, Spanish. doi: 10.5546/aap.2015.397. PMID: 26294143.	Investigations beyond the scope of primary care - Uses laboratory/radiographic parameters

This showed a number of predictors which remained significant in a multivariable model in Malawi. These were: age; positive malarial rapid diagnostic test; difficulty breathing; grunting; chesty cough; respiratory rate; intercostal recession; and wheeze. In STARWAVe, current asthma; age (<2 years); inter-/sub-costal recession; illness duration (<4 days); moderate to severe vomiting (within 24 hours of presenting); wheeze; and body temperature (>37.8 degrees celcius or parent-reported severe fever within 24 hours of presenting) were the predictors employed by the final model.

Interestingly, in both the STARWAVe study's cohort and the BIOTOPE cohort, oxygen saturation did not remain predictive in multivariable models, but was predictive in univariate analysis. Analysis of investigations like pulse-oximetry in such cohorts assists with decision-making with regard to their implementation to routine primary care. There was no child with a diagnosis of asthma in the BIOTOPE cohort, this may be due to under-diagnosis, and may have impacted the results of the model performance. Whilst wheeze was predictive in both cohorts, illness duration, vomiting, and body temperature were not significant in the multivariable model when applied to the BIOTOPE cohort. Vomiting and body temperature are key symptoms of malarial illness, and its prevalence in Malawi may have led to loss of these predictors in the final model – this highlights the requirement for adaptability of models, to account for local factors.

## 4 Discussion

This study sought to conduct a review of all existing prediction models for hospitalisation due to lower respiratory tract infection (LRTI) in children aged 2-59 months used in primary care and the subsequent external validation of these models in a novel cohort of Malawian paediatric patients (BIOTOPE Cohort).

Only one rule was identified and it had undergone derivation and internal validation. It used hospitalisation within 30 days as a surrogate outcome for the need for antibiotic prescription [13]. On external validation in the BIOTOPE cohort, a similar AUC for hospitalisation was found (0.81 in STARWAVe and 0.8 in BIOTOPE) but a much lower AUC for bacterial pneumonia as defined in BIOTOPE (0.39 in BIOTOPE). No microbiological determination of bacterial infection was undertaken in the STARWAVe study with hospitalisation within 30 days used as a substitute measure of appropriate antibiotic prescription. However, previous studies have shown that hospitalisation does not necessarily correlate with bacterial infection, with a study conducted in the USA reporting that only 8% of children hospitalised with suspected bacterial pneumonia were found to have an infection of bacterial aetiology [14]. Although defining bacterial aetiology is challenging in lower respiratory tract infection without the use of invasive procedures, host response biomarkers may provide a useful tool and studies are ongoing in this area, such as the BIOTOPE study.

In the STARWAVe study, 750 of the children included in the study have a concomitant diagnosis of asthma, comprising almost 10% of the sample population. Asthma was one of the predictor parameters associated with hospital admission and further work is required to determine the accuracy of this rule in a population of non-asthmatic patients. In BIOTOPE, no child was on medication for wheeze at home, nor had any child a previous diagnosis of asthma - this demonstrates the broad applicability of this rule in a non-asthmatic population also [11]

The statistical model used to develop the checklist (STARWAVe) involved the inclusion of children who received antibiotics (37% of the study sample). This limits the value of the checklist as it is not representative of the risk of hospitalisation in those patients not receiving antibiotic therapy. However, in BIOTOPE, all children were enrolled prior to their first prescription of antibiotics. Given the similar performance of the rule in the BIOTOPE cohort to predict hospitalisation, this does not appear to influence the predictive ability of the rule.

Antibiotic resistance is rapidly evolving into one of the greatest challenges faced by primary care physicians. At present, WHO Guidelines advise that all children in the paediatric population specified in our study, with suspected bacterial pneumonia should be treated with antibiotics [12]. Given the recent advancements in both the development and international availability of vaccinations and their impact on infection, this may no longer be the most appropriate approach. The introduction of malaria rapid-diagnostic tests to primary care in endemic countries has led to a significant reduction in the use of antimalarials, but at the expense of increased antibiotic prescribing [15,16]. This highlights the need to develop appropriate strategies for diagnosing bacterial LRTI in primary care. Since hospitalisation may not represent bacterial aetiology, strategies will need to be developed to identify those with such aetiology. However, even with this, there will also be a need to identify those at risk of severe disease so that they can be appropriately referred to hospital. The BIOTOPE study showed that WHO severity criteria were present in a minority (30.4%) of children hospitalised with pneumonia, consistent with recent work in the Lancet Global Health, showing that 39% of fatal cases of pneumonia were defined as having non-severe pneumonia, requiring only home treatment by the 2013 revision [11,17]. The fact that the WHO criteria would have discharged these children with oral antibiotics accentuates the need for new markers of severity, the STARWAVe rule may serve as a useful tool in this regard [13].

This systematic review of clinical prediction rules for hospitalisation due to lower respiratory tract infection in children in primary care made use of an extensive literature search and standardised critical appraisal of the model using the CHARMS checklist of the Cochrane Collaboration [9].

The validation cohort in this study is small relative to that of the cohort used to derive the STARWAVe rule. The BIOTOPE Cohort (n=494), though smaller, yielded a similar number of hospitalisations as that of the STARWAVe group (56 in BIOTOPE; 78 in STARWAVe). This may be attributed to BIOTOPE enrolling only children who had WHO defined pneumonia and the lower socioeconomic status of Malawi influencing illness severity. It is important to note that while the rule was developed in the United Kingdom, a country in which general practitioners act as the primary point of access to a health service with specialist referral possible, our validation study was conducted in Malawi – a location wherein primary care is oftentimes the only form of healthcare available to the population, thereby confirming the global applicability of the STARWAVe rule across a variety of healthcare settings.

There is no single internationally accepted definition of a clinical prediction rule. This resulted in a limitation to our own work as we had to pre-determine a single definition to facilitate the screening process in this systematic review.

There were no children with a diagnosis of asthma in the BIOTOPE cohort. This unfortunately may be attributable to under-diagnosis and may impact on the results of the model's performance

Systematic reviews should serve to guide evidence-based decision making, leading to recommendations on which models to integrate into guidelines, in this instance, for the diagnosis of lower respiratory tract infection in children. Given the lack of clinical prediction rules available for predicting hospitalisation from LRTI specifically for this population and the lack of clinical application studies for these rules, it is difficult to measure the impact they have on patient care, physician behaviour and healthcare costs. There have been reports of large numbers of clinical prediction rules being developed without sufficient validation and clinical impact studies leading to calls for refinement of existing clinical prediction rules, rather than the development of more [18]. However, in this case, there was only one rule found. It performed well in predicting hospitalisation in both cohorts, but further work on differentiating bacterial from viral aetiology needs to be undertaken, with consideration given to the integration of point-of-care testing as it becomes available. If the implementation of the STARWAVe tool leads to an elevated rate of antibiotic prescription in high-risk patients, with a concurrent decrease in prescriptions in those at low risk, it could achieve a 10% reduction in antibiotic prescriptions for respiratory tract infections in primary care [13]. This reduction could have a significant impact on the development of antibiotic resistance, on a global scale. The high accuracy of the STARWAVe model in the Malawian BIOTOPE cohort was primarily attributable to high specificity. It has been suggested that a model with a higher sensitivity may be more appropriate in this setting, ensuring that false negatives are limited and children are more appropriately referred to hospital centres [19]. This concurs with the concerns raised in the study by Agweyu et al. showing that 39% of fatal pneumonia cases were defined as having non-severe pneumonia, requiring only home therapy, by the 2013 WHO revision [17].

The use of clinical prediction rules in primary care for the prediction of hospitalisation as a consequence of lower respiratory tract infection could facilitate the advancement of antibiotic stewardship by providing clinicians with a tool to reduce clinical uncertainty, thereby decreasing the administration of antibiotics to children who are at low-risk for hospitalisation. They can also be employed to guide clinicians in appropriate referral-making to secondary care. Often, in lower income settings, there is limited transport infrastructure and significant distances between patients and their nearest secondary centre. There is a lack of clinical prediction rules in this area. The STARWAVe rule identified in this review was a good tool for predicting hospitalisation but not for bacterial infection as defined. It had a low sensitivity, and clinical impact studies are required to ensure that children are referred to hospital appropriately, without overburdening secondary care systems with inappropriate referrals. The STARWAVe rule did not perform as well as microbiological methods for identifying bacterial aetiology However, in the absence of a gold standard indicator for bacterial LRTI this could be considered a reasonable surrogate and could lead to significant reductions in antibiotic prescription rates should clinical impact studies prove its utility. Further work to determine its clinical impact and to identify better ways of diagnosing bacterial LRTI in primary care are required.

**Table:** 

EMBASE	Search Terms
1	(‘respiratory tract infecton/’exp OR ‘respiratory tract infecton’ OR ‘respiratory infection*’ OR rti OR lrti OR lri OR ‘chest infection*’ OR ‘cough’/exp OR cough OR ‘dyspnoea’ OR ‘congestion’/exp OR ‘lung consolidation’/exp OR ‘pneumonia’/exp OR pneumonia OR ‘difficult breath*’ OR ‘respiration disorder*’) AND [1966-2021]/py
2	(child* OR schoolchild* OR preschool* OR paediatric* OR paediatric* OR infant OR ‘infancy’
3	(Model* OR Predict* OR Decision*or) AND score* OR rule*
4	(primary AND care OR family) AND practice or general) AND practice OR family) AND medicine OR community) AND healthcare OR primary) AND healthcare OR ambulatory) AND healthcare
5	#1 and #2
6	#4 and #5
7	#3 and #6
*	The asterisk (*) represents any group of characters, including no character
""	Only finds articles with this phrase

## Funding

This study was funded in whole or in part by the support of the Bill & Melinda Gates Foundation (Investment ID: OPP1139557). The foundation was not involved in study design, data collection, analysis, interpretation or drafting of this report.

## Contributors

Contributors DW and JG conceived the review, developed the search strategy and drafted the manuscript. ML performed data analysis and interpretation. MC, PH, RJD and CW assisted with study design, analysis and interpretation of data. JG, ML, CW were responsible for the raw data utilised in this study. All authors contributed to manuscript development, edited for critical content, and have approved the final version.

## Data sharing statement

Study data pertaining to the BIOTOPE Cohort, may be made available upon reasonable request from the corresponding author.

## Declaration of Competing Interest

All authors declare that they have no conflicting or competing interests of a financial nature or otherwise.

## Appendix Supplementary materials

Image, application 1

## References

[1] O'Brien Katherine L., Baggett Henry C., Abdullah Brooks W., Feikin Daniel R., Hammitt Laura L., Howie Stephen R.C., Knoll Maria Deloria, Kotloff Karen L., Levine Orin S., Madhi Shabir A., Murdoch David R., Anthony G. Scott J., Thea Donald M., Zeger; Scott L. (15 June 2017). Introduction to the Epidemiologic Considerations, Analytic Methods, and Foundational Results From the Pneumonia Etiology Research for Child Health Study. Clinical Infectious Diseases.

[2] Butler CC, Rollnick S, Pill R, Maggs-Rapport F, Stott N. (1998). Understanding the culture of prescribing: qualitative study of general practitioners' and patients' perceptions of antibiotics for sore throats. BMJ.

[3] Cabral C, Lucas PJ, Ingram J, Hay AD, Horwood J. (2015). It's safer to …” parent consulting and clinician antibiotic prescribing decisions for children with respiratory tract infections: an analysis across four qualitative studies. Soc Sci Med.

[4] (2017). Public Health England English surveillance programme for antimicrobial utilisation and resistance (ESPAUR). Report.

[5] Fairall L, Bateman E, Cornick R (2015). et alInnovating to improve primary care in less developed countries: towards a global model. BMJ Innovations.

[6] McGinn TG, Guyatt GH, Wyer PC, Naylor CD, Stiell IG, Richardson WS. (2000 Jul 5). Users' guides to the medical literature: XXII: how to use articles about clinical decision rules. Evidence-Based Medicine Working Group. Jama.

[7] Fahey T, Van der Lei J., Knottnerus A, Buntinx F (2008). Evidence base of clinical diagnosis.

[8] Moher D, Liberati A, Tetzlaff J, Altman DG, Group P. (2009 Jul 21). Preferred reporting items for systematic reviews and meta-analyses: the PRISMA statement. PLoS Med.

[9] Moons KGM, de Groot JAH, Bouwmeester W, Vergouwe Y, Mallett S (2014). Critical Appraisal and Data Extraction for Systematic Reviews of Prediction Modelling Studies: The CHARMS Checklist. PLoS Med.

[10] Keogh C. (2014). Developing an international register of clinical prediction rules for use in primary care: a descriptive analysis. Ann Fam Med.

[11] Gallagher J, Chisale M, Das S (2021). On behalf of BIOTOPE team, et al Aetiology and severity of childhood pneumonia in primary care in Malawi: a cohort study. BMJ Open.

[12] Recommendations for management of common childhood conditions, Evidence for technical update of pocket book recommendations. Geneva: World Health Organization; 2012 (http://www.who.int/maternal_child_adolescent/documents/management_childhood_conditions/en). 23720866

[13] Hay, A. D., et al. "Development and internal validation of a clinical rule to improve antibiotic use in children presenting to primary care with acute respiratory tract infection and cough: a prognostic cohort study." The Lancet Respiratory Medicine 4(11): 902-910. 10.1016/S2213-2600(16)30223-5 PMC5080970 27594440

[14] Jain S, Williams DJ, Arnold SR (2015). Community-Acquired Pneumonia Requiring Hospitalization among U.S. Children. The New England journal of medicine.

[15] Odaga J, Sinclair D, Lokong JA, Donegan S, Hopkins H, Garner P. (2014). Rapid diagnostic tests versus clinical diagnosis for managing people with fever in malaria endemic settings. Cochrane Database Syst Rev.

[16] Heidi Hopkins, J Bruxvoort Katia, E Cairns Matthew, R Chandler Clare I, Baptiste Leurent, Ansah Evelyn K (2017). Impact of introduction of rapid diagnostic tests for malaria on antibiotic prescribing: analysis of observational and randomised studies in public and private healthcare settings. BMJ.

[17] G Damen Johanna A A, Lotty Hooft, Ewoud Schuit, A Debray Thomas P, S Collins Gary, al Tzoulaki Ioannaet (2016). Prediction models for cardiovascular disease risk in the general population: systematic review. BMJ.

[18] Agweyu A, Lilford RJ, English M (Jan 2018). Clinical Information Network Author G. Appropriateness of clinical severity classification of new WHO childhood pneumonia guidance: a multi-hospital, retrospective, cohort study. The Lancet Global health.

[19] Keogh C, Fahey T, Guest Editorial (5 October 2010). Clinical prediction rules in primary care: what can be done to maximise their implementation?. Clinical Evidence.

