# Supplementary material for: A Systematic Review of Clinical Prediction Rules to Predict Hospitalisation in Children with Lower Respiratory Infection in Primary Care and their Validation in a New Cohort
Source: eClinicalMedicine. 2021 Oct 18;41:101164. doi: 10.1016/j.eclinm.2021.101164 (PMC8529204; doi:10.1016/j.eclinm.2021.101164)
Supplement: Supplementary file 1 [file mmc1.docx]

Supplemental File

**A Systematic Review of Clinical Prediction Rules to Predict Hospitalisation from Lower Respiratory Infection in Children in Primary Care and their Validation in a New Cohort**

Table of Contents

[S1: Diagnostic criteria for bacterial and viral pneumonia 2](#_Toc82010869)

[S2 Logistic regression model of markers of severity 2](#_Toc82010870)

[Figure S1 Logistic regression model calibration curves 4](#_Toc82010871)

[S3: Demographics of cohorts used in STARWAVe & BIOTOPE studies: 5](#_Toc82010872)

## S1: Diagnostic criteria for bacterial and viral pneumonia

| **Group** | **Criteria** |
| --- | --- |
| **Bacterial pneumonia** | Positive blood culture for significant pathogen (e.g. S. pneumoniae, S.aureus, H. influenzae)  Or  H.influenzae/ S.aureus/ S. pneumoniae detected by blood PCR  With or without  virus detected by RT-PCR |
| **Viral pneumonia** | Negative blood culture for significant pathogen (e.g. S. pneumoniae, S.aureus, H. influenzae,)  AND  H.influenzae/ S.aureus / S. pneumoniae not detected by blood PCR  With  Virus detected by RT-PCR |
| **Unknown etiology** | Negative blood culture for significant pathogen (e.g. S. pneumoniae, S.aureus, H. influenzae)  AND  H.influenzae/ S.aureus/ S. pneumoniae not detected by blood PCR  Without  Virus detected by RT-PCR |

## S2 Logistic regression model of markers of severity

Using the logistic regression model the following was found for hospitalisation

| **Variable** | **Univariate** | **Full model** | **Step model** |
| --- | --- | --- | --- |
| Age in months (log transformed) | -0.624*** | -0.545* | -0.475* |
| Male | 0.018 | -0.199 | - |
| Malnourished | -14.496 | -15.2 | - |
| History of prematurity | 0.884* | 0.533 | - |
| WHO defined severe pneumonia | 1.367*** | 0.533 | - |
| Previous pneumonia admission | 0.227 | -0.665 | - |
| Malaria positive | 0.482 | 1.099 | 1.160* |
| Fever or history of fever | 0.335 | -1.494 | - |
| Difficulty breathing | 2.342*** | 1.550** | 1.465** |
| Vomiting | 0.905** | 0.675 | 0.812 |
| Diarrhoea | 0.25 | 0.53 | - |
| Coryza | 0.027 | -0.134 | - |
| Sneezing | -0.546 | -0.6 | -0.847 |
| Pulling at ears | 0.071 | -0.285 | - |
| Grunting | 2.340*** | 1.141 | 1.268* |
| Nasal flaring | 1.458*** | 0.088 | - |
| Deep breathing | 2.720*** | 1.833*** | 1.660*** |
| Lower chest wall indrawing | 2.222*** | 1.002 | 1.164* |
| Head nodding | 1.838*** | -1.597 | -0.972 |
| Wheeze | 1.471*** | 2.128*** | 2.098*** |
| Crackles | 0.906* | 0.997 | - |
| Dry cough | -0.551 | 14.273 | - |
| Chesty cough | 0.703* | 15.142 | 1.099* |
| Duration of cough | -0.14 | -0.473* | -0.334 |
| Tympanic temperature (^o^C) | 0.376** | 0.408 | - |
| Heart rate (bpm) | 0.017** | 0.012 | 0.013 |
| Respiratory rate (BPM) | 0.096*** | 0.059* | 0.065** |
| Oxygen saturation | 1.187** | 1.03 | 0.929 |
| Capillary refill time (seconds) | -0.503 | -0.755 | - |
| HIV positive | -0.223 | 0.59 | - |
|  |  |  |  |

**Internal validation using bootstrapping**

Results: AUC = 0.92 (0.90, 0.93), calibration slope = 0.79 (0.55, 1.04), Tjur R-sq = 48.8% (40.9, 56.7).

### Figure S1 Logistic regression model calibration curves

**Calibration curves are outlined below**

**
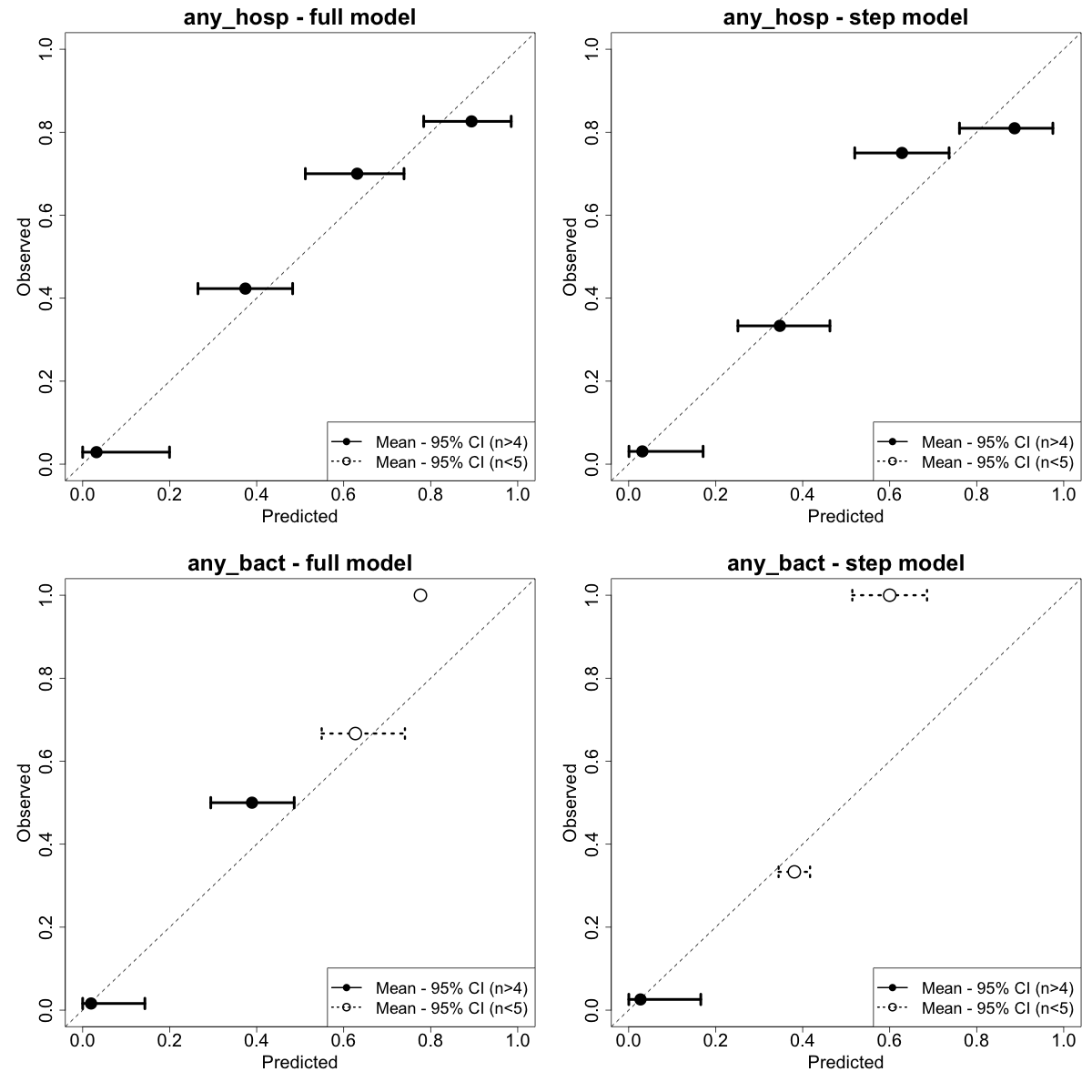
**

| **Model** | **AUC** | **AUC lower CI95** | **AUC upper CI95** | **Tjur Rsq** | **Threshold for Sens. 95%** | **Sensitivity at Sensitivity of 95%** | **Specificity at Sensitivity of 95%** |
| --- | --- | --- | --- | --- | --- | --- | --- |
| FULL | 0.95 | 0.93 | 0.97 | 51.3 | 0.089 | 0.95 (0.85, 0.99) | 0.85 (0.81, 0.89) |
| STEP | 0.93 | 0.91 | 0.96 | 48.6 | 0.046 | 0.95 (0.85, 0.99) | 0.73 (0.69, 0.78) |

## S3: Demographics of cohorts used in STARWAVe & BIOTOPE studies:

| **Parameter** | **BIOTOPE (Total)**  **n=494** | **BIOTOPE (Hospitalised)**  **n=56** | **STARWAVe**  **(Total)**  **n=8394** | **STARWAVe (Hospitalised)**  **n=78** |
| --- | --- | --- | --- | --- |
| Gender | 271 (54.8%) male | 31 (55.4%) male | 52% male | 51 (65%) |
| Median age | 1.5 years (IQR 0.83-2.5) | 1.1 years (IQR 0.5-1.75) | 3 years (IQR 1-6) | Not specified |
| Number recruited | 494 |  | 8394 |  |
| Number hospitalised | 56 |  | 78 [0.9%] C.I 0.7-1.2% |  |
| Number who received antibiotics | 442 (94.8%) | 52 (92.8%) | 3121 (37.2%) | 25 (32%) |
| Setting | Two primary care centres in Mzuzu, Malawi |  | 247 GP practices across England |  |
| Asthma | 0 | 0 | 750 (8.9%) | 19 (24%) |
| Vomiting in preceding 24 hours | 122 (24.7%) | 23 (41.1%) | 2347 (27.9%) | 20 (26%) |
| Inter/Sub-costal recession | 172 (34.8%) | 44 (78.6%) | 404 (4.8%) | 25 (32%) |
| Wheeze | 36 (7.3%) | 11 (19.6%) | 1236 (14.72%) | 34 (44%) |
| Temperature greater than/equal to 37.8 degrees | 470 (95.1% | 53 (94.6%) | 1046 (12.46%) | 20 (26%) |
